# A Case of Type II Achalasia Presenting With Markedly Elevated Troponins

**DOI:** 10.7759/cureus.33408

**Published:** 2023-01-05

**Authors:** Talia F Malik, Daniel Khan, Pallavi Shah, Andrew M Moon

**Affiliations:** 1 Department of Internal Medicine, Chicago Medical School at Rosalind Franklin University of Medicine and Science, North Chicago, USA; 2 Department of Gastroenterology, Captain James A. Lovell Federal Health Care Center, North Chicago, USA; 3 Department of Medicine, Division of Gastroenterology and Hepatology, University of North Carolina at Chapel Hill School of Medicine, Chapel Hill, USA

**Keywords:** acute coronary syndrome, megaesophagus, myocardial injury, troponin, achalasia

## Abstract

Achalasia is an esophageal motility disorder that presents with dysphagia to solids and liquids and regurgitation of undigested food. Cardiac troponin (cTn) is a sensitive biomarker for myocardial injury, and elevated levels suggest an increased risk of mortality from acute coronary syndrome (ACS). Non-cardiac gastrointestinal (GI) causes of troponin elevation are rare and have generally been described in cases of critical illness (e.g., significant gastrointestinal bleeding (GIB) or acute liver failure). We report a rare case of type II achalasia presenting with markedly elevated troponins. This case illustrates an important GI-related mimic of ACS that should be considered by frontline providers and gastroenterologists.

## Introduction

Achalasia is an esophageal motility disorder characterized by incomplete relaxation of the lower esophageal sphincter (LES) and loss of peristalsis in the esophageal wall [1]. It arises from the progressive degeneration of ganglion cells in the myenteric plexus of the esophageal wall and LES. The resultant loss of inhibitory neurotransmitters leads to unopposed cholinergic activity causing incomplete LES relaxation. Esophageal dilation in achalasia results in the classic presentation of dysphagia to solids and liquids and regurgitation of undigested food or saliva. These symptoms are often accompanied by chest pain, weight loss, and heartburn [2]. We report a unique case of achalasia presenting with elevated cardiac troponins (cTn) prompting cardiac evaluation. This case reports on an important gastrointestinal (GI)-related cause of chest pain that should be included in the differential diagnosis, even with elevations of cTn.

This case was presented as a poster presentation at the American College of Gastroenterology Annual Scientific Meeting in Charlotte, North Carolina, on October 21-26, 2022.

## Case presentation

A 74-year-old female with a medical history of hyperlipidemia presented with diffuse aching chest pain and shortness of breath for one day. She complained of postprandial dysphagia to solids and liquids one day before the initial presentation. Physical examination revealed tachypnea and diffuse chest wall tenderness. Laboratory studies revealed markedly elevated serial troponin levels of 0.06 ng/mL, 0.09 ng/mL, and 1.14 ng/mL (reference value: 0-0.04 ng/mL). The Thrombolysis in Myocardial Infarction (TIMI) score was 2, suggesting an 8% risk of 14-day all-cause mortality, new or recurrent myocardial infarction, or recurrent severe ischemia. This prompted an emergent cardiac evaluation with an electrocardiogram (EKG) showing normal sinus rhythm, followed by a transthoracic echocardiogram (TTE) that did not reveal any abnormality.

**Figure 1:**
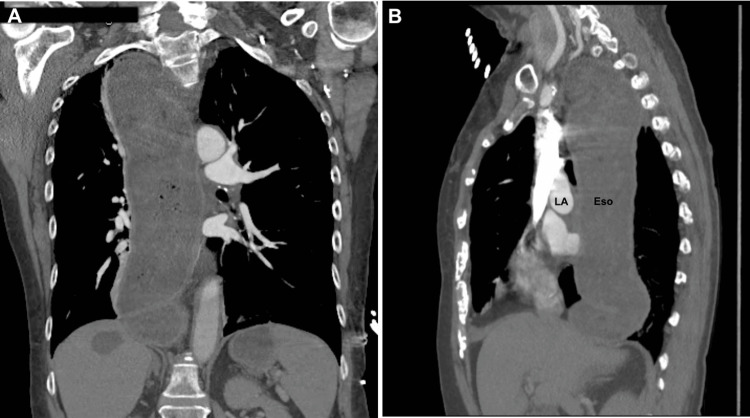
Computed tomography angiography of the chest and abdomen showing markedly dilated esophagus (Eso) against the left atrium (LA) in coronal (A) and sagittal (B) view. Eso: esophagus, LA: left atrium

**Figure 2:**
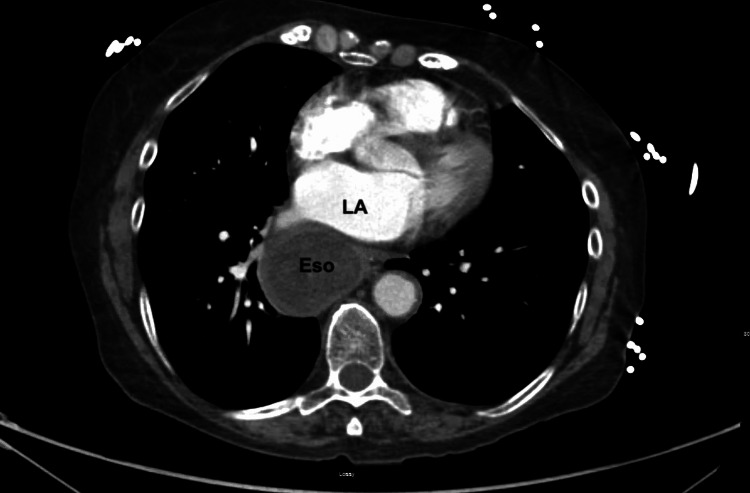
Computed tomography angiography of the chest and abdomen showing markedly dilated esophagus (Eso) against the left atrium (LA) in axial view. Eso: esophagus, LA: left atrium

Computed tomography angiogram of the chest and abdomen demonstrated a megaesophagus measuring 6 cm in diameter containing a large amount of debris extending to the cervical esophagus with marked narrowing of the gastroesophageal junction (Figure 1 and Figure 2).

**Figure 3:**
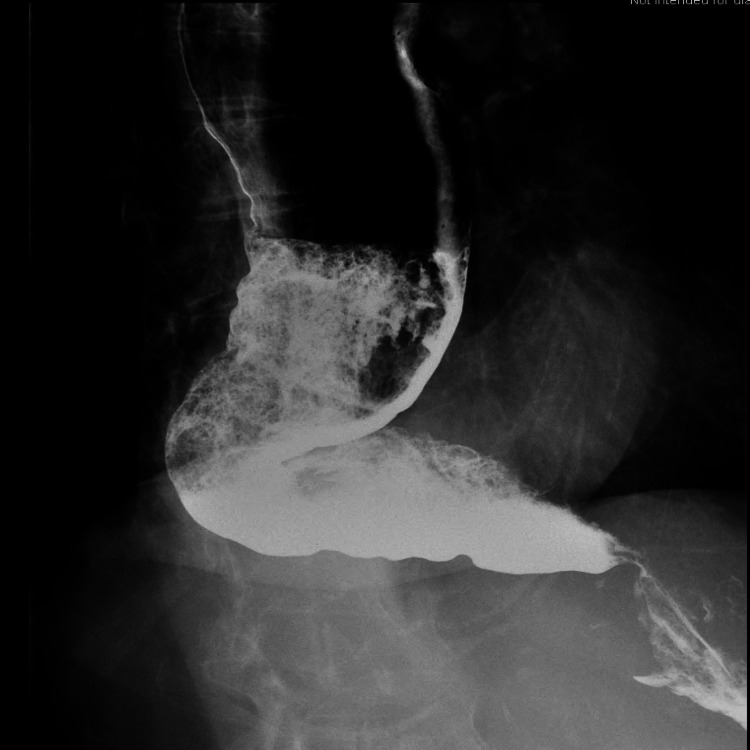
Barium esophagram showing dilated esophagus with narrowing at the gastroesophageal junction, demonstrating the classic “bird’s beak appearance.”

Barium esophagram showed a dilated esophagus with narrowing at the lower esophagus, also known as “bird’s beak appearance” (Figure 3).

**Figure 4:**
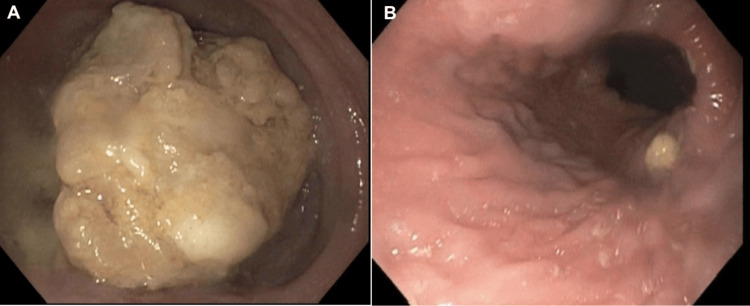
Esophagogastroduodenoscopy showing the esophageal lumen filled with undigested food and debris before (A) and after (B) removal.

A decision was made to not pursue further cardiac workup as elevated cTn was likely due to non-cardiac etiology in the setting of normal EKG and TTE. Esophagogastroduodenoscopy (EGD) revealed a severely dilated esophagus with large amounts of undigested food and hypertonic LES (Figure 4A). The undigested food was removed, and botulinum toxin was injected at the LES, followed by balloon dilation. No mass, strictures, or signs of external compression were seen (Figure 4B).

The patient’s symptoms subsided following the EGD, and repeat serial troponin levels showed a downward trend. The patient tolerated liquid diet and was discharged with instructions to advance diet as tolerated over the next few days. The patient was referred to a tertiary care center for further management. High-resolution esophageal manometry was done that showed elevated median integrated relaxation pressure, abnormal peristalsis, and panesophageal pressurization confirming the diagnosis of type II achalasia. She subsequently underwent an uncomplicated Heller myotomy with fundoplication with gradual improvement in symptoms over the course of several days. The patient reported complete resolution of symptoms at the one-month follow-up.

## Discussion

Troponins are the most specific and sensitive biomarkers of myocardial injury [3]. The American Heart Association/American College of Cardiology guidelines and European Society of Cardiology task force report have emphasized the significance of the utilization of troponin measurements and TIMI score for risk stratification in acute coronary syndrome (ACS) without ST-segment elevation [4,5]. Elevated cTn reflects irreversible myocyte necrosis, and markedly elevated troponin levels suggest acute thrombotic myocardial ischemia. Rarely, non-cardiac conditions that result in myocardial injury without myocardial ischemia may result in troponin elevation [3]. These conditions include pulmonary embolism, end-stage renal disease, sepsis, stroke, and critically ill patients [6]. In these conditions, cTn has been reported to predict mortality, and elevated levels have been associated with a worse prognosis [3,7,8].

Gastrointestinal causes of elevated troponins are rare and associated with increased mortality and morbidity in patients without ACS [9,10]. Acute gastrointestinal bleeding (GIB) and acute liver failure have been known to be associated with elevated troponins [10,11]. The underlying mechanism in these conditions is thought to be an increase in myocardial oxygen demand together with a decrease in myocardial oxygen supply by shock, tachycardia, and hypoxemia [8,11]. The resulting myocardial injury causes the release of cTn from cardiomyocytes. In achalasia, the likely mechanism of elevated troponins is postulated to be left atrial compression secondary to the enlarged esophagus resulting in myocardial damage and cTn release. Left atrial compression secondary to an enlarged esophagus is rare and reported to present as heart failure and hemodynamic instability and with symptoms of ACS without troponin elevation [12-15]. In our case, left atrial compression due to close anatomical proximity of the dilated esophagus and left atrium likely caused cTn release due to an increase in myocardial wall stress causing reduced subendocardial perfusion [16]. Interestingly, unlike previously reported cases, our patient was found to have elevations of cTn without apparent compression of the left atrium on TTE.

Achalasia is a rare condition, and an unusual presentation with markedly elevated troponins can present a diagnostic challenge. Interestingly, a case of achalasia presenting with elevated troponin has been reported in the literature; however, only mild troponin elevation (peak troponin level: 0.076 ng/dL) was noted compared to our case where the troponin levels were considerably high [17]. This case highlights the importance of forming a broad differential when evaluating patients presenting with elevated troponins. While cTn elevation is commonly associated with ACS, the complete clinical picture should be assessed, and all cardiac and non-cardiac causes of cTn elevation should be ruled out. In patients with acute GIB, elevated troponin on presentation is associated with higher short-term mortality and a longer hospital stay, which may also be true for achalasia [11]. Early diagnosis and prompt intervention are beneficial as cTn elevation in patients without ACS has been associated with a worse prognosis [7].

## Conclusions

In conclusion, achalasia may present with markedly elevated troponins with no underlying cardiac disease. Achalasia should be considered in the differential diagnosis of gastrointestinal causes of markedly elevated troponins once acute coronary syndrome has been ruled out. Frontline clinicians and gastroenterologists should be aware of this unusual presentation to prevent delayed diagnosis, avoid unnecessary interventions, and improve outcomes.
